# China’s practice to prevent and control COVID-19 in the context of large population movement

**DOI:** 10.1186/s40249-020-00716-0

**Published:** 2020-08-19

**Authors:** Tie-Long Xu, Mei-Ying Ao, Xu Zhou, Wei-Feng Zhu, He-Yun Nie, Jian-He Fang, Xin Sun, Bin Zheng, Xiao-Fan Chen

**Affiliations:** 1 Evidence-based Medicine Research Center, Jiangxi University of Traditional Chinese Medicine, Nanchang City, Jiangxi Province P. R. China; 2 Chinese Evidence-Based Medicine Center, West China Hospital, Sichuan University, Chengdu City, Sichuan Province P. R. China; 3 National Institute of Parasitic Diseases, Chinese Center for Disease Control and Prevention, and National Center for Tropical Diseases Research, Shanghai, People’s Republic of China

**Keywords:** COVID-19, SARS-CoV-2, Prevention and control, Endemic, Pandemic

## Abstract

**Background:**

The emerging infectious disease, coronavirus disease 2019 (COVID-19) caused by severe acute respiratory syndrome coronavirus 2 (SARS-CoV-2), poses a serious threat in China and worldwide. Challenged by this serious situation, China has taken many measures to contain its transmission. This study aims to systematically review and record these special and effective practices, in hope of benefiting for fighting against the ongoing worldwide pandemic.

**Methods:**

The measures taken by the governments was tracked and sorted on a daily basis from the websites of governmental authorities (e.g. National Health Commission of the People’s Republic of China). And the measures were reviewed and summarized by categorizations, figures and tables, showing an ever-changing process of combating with an emerging infectious disease. The population shift levels, daily local new diagnosed cases, daily mortality and daily local new cured cases were used for measuring the effect of the measures.

**Results:**

The practices could be categorized into active case surveillance, rapid case diagnosis and management, strict follow-up and quarantine of persons with close contacts, and issuance of guidance to help the public understand and adhere to control measures, plus prompt and effective high-level policy decision, complete activation of the public health system, and full involvement of the society. Along with the measures, the population shift levels, daily local new diagnosed cases, and mortality were decreased, and the daily local new cured cases were increased in China.

**Conclusions:**

China’s practices are effective in controlling transmission of SARS-CoV-2. Considering newly occurred situations (e.g. imported cases, work resumption), the control measures may be adjusted.

## Background

Coronavirus disease 2019 (COVID-19) is an emerging infectious disease caused by severe acute respiratory syndrome coronavirus 2 (SARS-CoV-2) [1, 2]. The emerging disease spreads fast through respiratory droplets or close contact [3, 4], with an estimated *R*_0_ of up to 5.7 (95% *CI*:3.8–8.9) [5]. People at any age are susceptible [6–10]. In some countries, the number of cases doubles every 3 to 4 days [11]. Moreover, It is estimated to be 10 times fatal than 2009 H1 N1 flu pandemic [11]. As of May 10, 2020, a total of 278 279 people was dead of COVID-19 in the world, and 1 339 071 people recovered, with a nominal mortality of 17.2%. A number of health workers were also infected with COVID-19, with over 10% of healthcare professionals being infected in some countries [12]. On March 11, 2020, the World Health Organization (WHO) announced COVID-19 a pandemic situation [13].

In China, COVID-19 epidemic began just before the Spring Festival [14–17], an event with the world largest population movement [18]. In such a situation, China has taken many special measures to control the virus transmission. Currently, the newly diagnosed cases per day in the mainland of China have dropped to only a few or even null [19]. However, the situation continues to be serious in other countries. Given the fact that COVID-19 pandemic is becoming more and more serious, we conduct this study to summarize the measures used in China, as well as the ultimate effect of the practices on SARS-CoV-2 transmission, with a hope to help others combating against the pandemic.

## Methods

### Data collection

The detailed data that we need is available at local official websites, especially the website of National Health Commission (NHC) of the People’s Republic of China. The relevant data reported from January 11 to June 6, 2020 about the epidemic in the mainland of China was tracked and sorted on a daily basis, including: 1) the policies or measures taken by the government; 2) official statistics about diagnosed cases, suspected cases, close contacts, deaths and recovered patients reported in a province or city. And the population shift levels, including inter-regional immigration index, inter-regional emigration indexes, intra-regional traffic intensity and overall Chinese population shift index were obtained from Baidu migration, a website focusing on the population shift during the Spring Festival [20]. Two groups collected the data separately and respectively, and conducted a cross check.

### Data analysis

Firstly, the policies or measures were systematically reviewed or described by nine items from the point of view of prevention and control, mainly involving the key responses at early emerging, case surveillance, case diagnosis and management, practice to cut off transmission route, and any other logistic services. Especially, the differences amongst versions of guidance for COVID-19 diagnosis and management, or, prevention and control were summarized and remarked, and official documents and some key actions were sorted by reported dates, so as to detailed record and illuminate China’s strategy to combat with the emerging infectious disease. Moreover, the latest prevention and control measures were abstracted from the latest guidance for COVID-19 diagnosis and management, or, prevention and control. Secondly, the population shift levels were plotted with dates and subject to period-over-period comparisons between 2019 and 2020, so as to display the whole effect of the practices on population shift. And the official statistics about case reports were used to calculate daily local new diagnosed cases, daily local new cured cases or mortality [deaths/(deaths + recovered patients) × 100%] of COVID-19, each of which was plotted with dates to induce the whole effect of the practices on the SARS-CoV-2 transmission.

## Results

## Measures or policies

### The key responses at early emerging

In the first few days, some important public health measures were implemented by authorities [21], mainly including: 1) reporting to the public about the unexplained viral pneumonia outbreak; 2) intensive surveillance and epidemiological investigations; 3) case finding, quarantine and management; 4) environmental sanitation and disinfection and closure of Huanan seafood market on January 1; 5) medical observation of close contacts to get evidence of human-to-human transmission; 6) public risk communication, efforts of improving public awareness and adoption of self-protection measures; 7) communication with WHO and other countries; 8) virus isolation and RNA sequencing, 9) establishing and sharing PCR detection kits.

### Active case surveillance

The possible patients were mainly from fever patients, close contacts, or suspected cases. Lots of fever clinic medical institutions were designated by the government to receive fever patients in each city. Body temperature monitoring stations for the floating population were set up at hubs of traffic, such as airport, station, wharf, expressway toll station, subway entrance, community entrance. Workers in enterprises, institutional units and government-affiliated institutions were required to do health declaration by employing units on a daily basis. Every city had specially designated fever clinic hospitals to identify suspected or confirmed cases, who might be quarantined for further diagnosis. Once a SARS-CoV-2 positive patient was confirmed, his/her close contacts would be found out for medical observation promptly. Impressively, the preceding travel routes of confirmed cases were announced to the public on official websites for self-discovery of the ones who were unconscious close contacts.

### Rapid case diagnosis and quarantine

#### Case diagnosis

The cases were comprised of kinds of suspected case, laboratory confirmed case, clinically diagnosed case (only for Hubei Province), death case, recovery case, mild, general, severe and critical type case, asymptomatic infection, and close contact. All the cases were diagnosed or cured according to the guidance for COVID-19 diagnosis and management [22].

To date, the guidance for COVID-19 diagnosis and management had been revised for seven times. All the seven versions covered the contact histories, clinical manifestations, diagnostic techniques, and manifestations of mild, general severe and critical types, and so on. The diagnostic criteria for each type of case based on each version was sorted, as shown in Additional file 2, in which the difference amongst versions was remarked by bold font. And the latest diagnostic criteria were summarized in Table 1. Firstly, the suspected case was judged and screened by combining the contact history and clinical manifestations. Secondly, the suspected case was further defined as laboratory confirmed case or clinically diagnosed case once the diagnostic criteria were met with. Thirdly, the confirmed or diagnosed cases needed to be further distinguished to be mild, general, severe and critical types. Besides, the diagnosis of close contact also followed the guidance for COVID-19 prevention and control (versions 1–6). As indicated in the latest versions, close contacts refer to people who have close contact with suspected, confirmed or diagnosed cases two-day before disease onset, or with asymptomatic carriers two-day before sampling but have not taken effective protection (Table 1, Additional file 2).

**Table 1:** The diagnosis and management in the latest guidance for COVID-19 prevention and control

Case type	Diagnosis	Management
Inspected case	V+(b + e)/V+(c + e)/V+(b + c)/(b + c + e)	Reporting in 2 h, Epidemiological survey in 24 h, Sampling and detection, Quarantine alone and treatment.
Laboratory diagnosed case	V+(b + e) + (5)/V+(c + e) + (5)/ V+(b + c) + (5)/ (b + c + e) + (5)	Reporting in 2 h, Epidemiological survey in 24 h, Quarantine alone and treatment.
Critical type	h/i/j	
Severe type	For adults: k/s/t/u, For children: v/w/x/y/z	
General type	b + e	
Mild type	e	
Recovery type	① + ②	Home isolation, self-health monitoring, and return visit at the 2nd and 4th week after discharge
Asymptomatic infection	(5)	Reporting in 2 h, Epidemiological survey in 24 h, Centered isolation for 14d with two times negative detection.
Close contact	iii	Home or centered isolation medical observation for 14 d.
Migrants from other cities or counties	Reported by themselves or quarantine workers	Home or centered isolation medical observation for 14 d.

Note: The meaning of each symbol is shown in the Additional file 2; “+” means “and”; “/” means “or”

It could be seen that the diagnostic criteria for each type of case were keeping updated with a clearer understanding of COVID-19, the epidemic progress and related technologies (Additional file 2).

#### Strict case quarantine and follow-up

These practices were intended for controlling infection sources, which might include suspected, confirmed or diagnosed cases, asymptomatic infection, and close contacts. All these cases were quarantined, cured, or medically observed according to the guidance for COVID-19 prevention and control [22]. The guidance had been revised six times with the updating knowledge about the endemic, and the ways for the case management were also continually perfected, as shown in Additional file 1: Table S1, in which the differences amongst versions were remarked by bold font. And the latest case management was sorted in Table 1. Besides, the management of recovered patients also followed the guidance for COVID-19 diagnosis and management (versions 1–7). As indicated in the versions 1–5, the recovered patients were discharged from hospitals without any suggestion, while in versions 6–7, they were suggested to do home isolation, self-health monitoring, and return visit on the 2nd and 4th week after discharge.

It could be seen that the case quarantine or follow up about each type of case were also keeping specified and improved with a clearer understanding of COVID-19, the epidemic progress and related technologies (Additional file 1: Table S1).

### Full involvement of the society to cut off transmission route

Except for the persons living in remote mountain areas, almost all people wore facemasks in public places. After being informed of human-to-human transmission, the public paid more attention to self-protection, and more health promotions and health educations were established to instruct the public on the correct way of wearing a mask and improving self-protection ability. On the night of January 22, 2020, it was decided to close outbound traffic of Wuhan City since January 23, which was imitated by other cities in Hubei Province. As of January 27, another 17 cities in Hubei Province were also closed, with only one city, Shennongjia, left unlocked. Although other cities in China did not take the same action, people in those places were confined to their homes. Initially, they were suggested to stay at home and were put under 24 h closed-off community management later. Specifically, one household could send one person to go out for shopping daily necessities every two or three days, and others could not go out unless a certification issued by relevant destination agencies could be presented. In some communities (e.g. Fuxingcheng Community in Xianning City, Hubei Province), none was permitted to go out and they were provided with daily necessities by government-assigning personnel. Community organizations were fully mobilized. Community management mainly involved body temperature measuring, case report, personnel follow-up, personnel access recording, home isolation.

All forms of gathering and meeting were prohibited, including working, visiting, and school activities. In view of this, the Spring Festival (Chinese New Year) holiday was extended from January 30 to February 2, but it went on at much reduced attendees. Work-resumption and schools reopening dates were also postponed, which varied from place to place with the situation of local epidemic (Table 2), and were carefully scheduled in different regions and units, so as to avoid the peak of passenger flow. Cultural and tourism exchange activities were not recommended and were subject approval by the local leading group for COVID-19 prevention and control and since January 24, 2020, all group tours were suspended in China. Schools canceled all forms of examination. Online training, teaching, communication and discussion were recommended, to avoid gathering as far as possible.

**Table 2:** Time points of some key actions in the mainland of China

Area	First case diagnosed	Emergency response timing sequence					Work resuming^&^	School reopening^&, #^					
		Level 2	Level 1	Level 2	Level 3	Level 4		Junior middle school	Senior middle school	Primary school	kindergarten	University	Vocational school
Hubei	Jan. 10	Jan. 22	Jan. 24	May 2	-	-	Feb. 13	May 6	Mid–May	-	-	-	-
Tibet	Jan. 28	Jan. 27	Jan. 29	Mar. 6	-	-	Feb. 2	Mar. 21	Mar. 15	-	-	-	-
Qinghai	Jan. 25		Jan. 25		Feb. 26	Mar. 6	Feb. 2	Mar. 16	Mar. 9	-	-	Apr. 1	Mar. 9
Xinjiang	Jan. 23		Jan. 25	Feb. 25	Mar. 7	Mar.21	Feb. 2	Mar.16	Mar. 16	Apr. 8	-	Apr. 8	Mar. 16
Ningxia	Jan. 22		Jan. 25	Feb. 28	May 6	-	Feb. 2	Mar. 25	Mar. 25	May 27	-	May 8	May 8
Neimenggu	Jan. 23		Jan. 25		Feb. 25	-	Feb. 9	Mar. 30	Mar. 30	May 7	-	May 7	May 7
Jilin	Jan. 22		Jan. 25	Feb. 26	Mar. 20	-	Feb. 2	Apr. 7	Apr. 7	June 1	-	May 7	-
Gansu	Jan. 23		Jan. 25		Feb. 21	May 11	Feb. 2	Apr. 13	Apr. 9	Apr. 13	May 25	Apr. 13	Apr. 9
Guizhou	Jan. 22		Jan. 24		Feb. 23	-	Feb. 2	Mar. 16	Mar. 16	May 25	May 25	Apr. 21	Apr. 21
Tianjin	Jan. 21		Jan. 24	Apr.30	June 6	-	Feb. 2	Apr. 20	Apr. 20	May 18	June 2	May 15	May 15
Liaoning	Jan. 22		Jan. 25		Feb. 22	-	Feb. 2	Apr. 15	Apr. 15	May 18	-	May 8	-
Shanxi*	Jan. 22		Jan. 25	Feb. 24	Mar. 10	-	Feb. 2	Mar. 25	Apr. 25	May 18	June 2	Apr.10	Apr. 25
Hainan	Jan. 23	Jan. 24	Jan. 25		Feb. 26	-	Feb. 2	Apr. 7	Apr. 7	Apr. 20	May 13	May 9	Apr. 13
Yunnan	Jan. 21		Jan. 24		Feb. 24	-	Feb. 9	Mar. 23	Mar. 23	Apr. 26	May 25	May 6	May 6
Hebei	Jan. 22		Jan. 24	Apr. 30	June 6	-	Feb. 2	Apr. 23	Apr. 23	June 1	June 8	June 6	June 1
Shanxi	Jan. 23		Jan. 25		Feb. 28	-	Feb. 2	Mar. 30	Apr. 7	Apr.27	June 8	Apr. 27	Apr. 7
Guangxi	Jan. 22		Jan. 24		Feb. 24	-	Feb. 2	Apr. 7	Apr. 7	May 6	May 25	Apr. 22	Apr. 7
Fujian	Jan. 22		Jan. 24	Feb. 26	Mar. 19	-	Feb. 9	Apr. 7	Apr. 7	May 11	May 20	May 6	May 6
Heilongjiang	Jan. 22		Jan. 25	Mar. 4	Mar. 25	-	Feb. 2	Apr. 13	Apr. 7	June 20	-	June 1	-
Shanghai	Jan. 20		Jan. 24	Mar. 23	May. 9	-	Feb. 9	Apr. 27	Apr. 27	June 2	June 2	Apr. 27	-
Beijing	Jan. 20		Jan. 24	Apr. 30	June 6	-	Feb. 9	Apr. 27	June 1	June 1	June 8	June 6	June 6
Sichuan	Jan. 21		Jan. 24	Feb. 26	Mar. 25	-	Feb. 2	Apr. 1	Apr. 7	June 2	June 2	May 16	Apr. 7
Shandong	Jan. 21		Jan. 24	Mar. 7	May. 6	-	Feb. 9	Apr. 15	Apr. 15	May 18	Early–June	May 18	Apr. 15
Jiangsu	Jan. 22		Jan. 24	Feb. 24	Mar. 27	-	Feb. 9	Mar. 30	Mar. 30	Apr. 7	-	Apr 13	Apr. 7
Chongqing	Jan. 21		Jan. 24	Mar. 10	Mar. 24	-	Feb. 9	Apr. 20	Apr. 20	Apr. 27	-	May 11	Apr. 20
Anhui	Jan. 22		Jan. 24	Feb. 25	Mar. 15	-	Feb. 9	Apr. 13	Apr. 7	Apr. 20	May 11	May 6	Apr. 20
Jiangxi	Jan. 21		Jan. 24	Mar. 12	Mar. 20	-	Feb. 9	Apr. 7	Apr. 7	May 18	May 25	Apr.23	Apr. 7
Hunan	Jan. 21		Jan. 24	Mar. 10	Apr. 1	-	Feb. 9	Apr. 7	Apr. 7	-	-	Mid–May	Mid–May
Henan	Jan. 21		Jan. 25	Mar. 19	May 6	-	Feb. 2	Apr. 7	Apr. 7	May 10	May 25	May 10	May 10
Guangdong	Jan. 19		Jan. 23	Feb. 24	May 9	-	Feb. 9	Apr. 27	Apr. 27	May 11	June 2	May 11	May 18
Zhejiang	Jan. 21		Jan. 23	Mar. 2	Mar. 23	-	Feb. 9	Apr. 13	Apr. 13	May 6	Mid–May	Apr. 26	Apr. 26

Note: “*”: is the province geographically near the Hebei province, “-”: the dates are still not fixed or not collected, “&”: except for some important enterprises related to national economy and people’s livelihood, the actual work resuming dates mustn’t be early than the fixed date or the actual school reopening dates were not early than the fixed date. And the fixed date here is just for reference. Local governments need to detail the arrangements to avoid the peak of return passenger flow. Generally, for school, graduates first, senior students second and then other students in each kind of school

The Civil Aviation Administration of China had cut the number of flights to Hubei Province since January 23, 2020, and until March 28, 2020, it was stated to resume domestic passenger flights to airports in Hubei Province except the Wuhan Tianhe Airport. Disinfection was regularly implemented in public places, grocery/vegetable/food markets, enterprises of catering, vehicles, residential areas, streets, and so on. Moreover, special groups (pregnant women, children, elderly people), special purpose entity (SPE) (e.g. school, hospital, children welfare office, prison, nursing home), and some special items (e.g. medical waste) were paid special attention with work regulations, schemes or notices issued by the government (Table 3).

**Table 3:** Key documents published on COVID-19 prevention and control

NO.	Title	Issued date
1	Law of the People’s Republic of China on Frontier Health Quarantine Inspection (revised for COVID-19)	Jan. 20
2	Law of the People’s Republic of China on the Prevention and Treatment of Infectious Diseases (revised for COVID-19)	Jan. 20
3	Guidance for COVID-19 prevention and control (Version 1)	unclear
4	Guidance for COVID-19 prevention and control (Version 2)	Jan. 22
5	Guidance for COVID-19 prevention and control (Version 3)	Jan. 28
6	Guidance for COVID-19 prevention and control (Version 4)	Feb. 7
7	Guidance for COVID-19 prevention and control Version 5)	Feb. 21
8	Guidance for COVID-19 prevention and control Version 6)	Mar. 7
9	Guidance for COVID-19 diagnosis and management (Version 1)	Jan. 16
10	Guidance for COVID-19 diagnosis and management (Version 2)	Jan. 18
11	Guidance for COVID-19 diagnosis and management (Version 3)	Jan. 23
12	Guidance for COVID-19 diagnosis and management (Version 4)	Jan. 27
13	Guidance for COVID-19 diagnosis and management (Version 5)	Feb. 5
14	Guidance for COVID-19 diagnosis and management (Version 6)	Feb. 18
15	Guidance for COVID-19 diagnosis and management (Version 7)	Mar. 3
16	The diagnosis and treatment scheme for severe and critical cases with COVID-19	Jan. 23
17	The control and prevention technical guide for COVID-19 in medical institutions (Version 1)	Jan. 23
18	Laboratory biosafety guidelines for SARS-CoV-2 (Version 2)	Jan. 23
19	Technical guidelines for selection and use of masks to prevent COVID-19 for different populations	Feb. 5
20	Prevention and control plan for SARS-COV-2 infection in community	Jan. 25
21	National Wildlife Trade Ban before the end of the epidemic	Jan. 25
22	Guiding principles of emergency psychological crisis intervention in the COVID-19 endemic	Jan. 26
23	Guidelines for the use limit of common medical protection equipment in the COVID-19 control	Jan. 27
24	The work scheme to transit patients with COVID-19	Jan. 28
25	Discharge standard for patients recovered from COVID-19	Jan. 28
26	Guidelines for SARS-CoV-2 infection prevention and control work in the children’s welfare service sector (Version 1)	Jan. 28
27	Management and technical guidelines for disposing of medical waste during COVID-19 endemic	Jan. 29
28	Operation technical guidelines for disinfection of public transports	Jan. 29
29	Health protection guidelines for COVID-19 in the public places	Jan. 30
30	Protection guidelines for different risk groups to avoid SARS-CoV-2 infection	Jan. 30
31	Use guidelines of gauze masks to prevent COVID-19	Jan. 30
32	Measures to improve the working conditions of front-line medical staff, care for the physical and mental health of medical staff	Jan. 30
33	Work guidelines for dealing with patient remains died of COVID-19	Feb. 1
34	Work schedule for medical observation at the isolation point of first diagnosis on mild suspected cases with COVID	Feb. 4
35	Guidelines for medical observation of infection by home isolation during COVID-19 prevention and control	Feb. 5
36	Guidelines for COVID-19 prevention and control during the epidemic in retail and catering enterprises	Feb. 6
37	The supporting policies for enterprises to resume work and production, combined with fighting against COVID-19 in Guangdong Province	Feb. 6
38	guidelines for COVID-9 prevention and control in nursing homes	Feb. 7
39	The suggestion for stabilizing labor relations and supporting enterprises to return to work and production	Feb. 7
40	Work guidelines for the psychological assistance hotline during the COVID-19 endemic	Feb. 7
41	Notice on strengthening the scientific prevention and control of COVID-19, combined with doing well the work of enterprise resumption	Feb. 8
42	Design guidelines for the emergency control and treatment facilities of COVID-19	Feb. 8
43	Operation management guidelines for the office and public places’ air conditioning and ventilation system during the COVID-19 endemic	Feb. 12
44	Health protection guidelines to avoid COVID-19 in Shopping malls and supermarkets	Feb. 14
45	User guideline of disinfectant	Feb. 18
46	Guidelines for prevention and control measures for enterprises and institutions returning to work in China	Feb. 22
47	Notice about scientific prevention and control and precise policy implementation, based on zoning and grading of endemic in different areas, so as to improve medical service.	Feb. 26

Note: the lists covered the releases by National Health CommitteeH from Jan. 20 to May 7, representing Chinese policy orientation according to different stages of endemic

In all, the measures for cutting off transmission route mainly consist of “lockdown” of city, extending the Spring Festival holiday, postponing the reopening of schools, suspending group tours, closing scenic spots, canceling mass gatherings, making fewer trips outsides, halting long-distance buses and reducing the frequencies of bus services so on.

### Effective high-level policy decision organization systems

During the fighting against SARS-CoV-2, governments at all levels have set up leading groups of central committees for COVID-19 prevention and control, holding the principle of giving priority to people’s life safety and health. During the epidemic, every important prevention and control activity, policy or measures were promptly designed or subject to approval by the local leading group.

### Full involvement of the society to guarantee manpower and material resources

#### Hospital preparation and construction

Besides the policies or measures for technology, manpower and material resources were also well organized by the government. During the epidemic, many fever clinic medical institutions, designated hospitals, isolation hospitals or points were prepared for COVID-19 patients, so as to implement observation, diagnosis, quarantine or treatment. Moreover, in Wuhan which suffered severe epidemic situation, two medical institutions (respectively named “Huoshenshan” and “Leishenshan”) were specially built within fourteen days or so with incredible efficiency and put into use since early February, playing an important role at the early stage of the epidemic. Since February 3, the Wuhan government had built “Fangcang hospitals” one by one. As of February 25, 2020, by means of house requisition, alteration and expansion, a total of 30 000 sickbeds in designated hospitals, 30 000 sickbeds in “Fangcang hospitals”, 10 000 sickbeds in isolation treatment points, and 8000 sickbeds in isolation observation stations were provided in readiness in Wuhan, solving the shortage of sickbeds.

#### Goods and materials

Undoubtedly, Hubei Province badly lacked goods and materials during the disease prevention and control, especially medical consumables and protective equipment. As early as January 22, 2020, National State-owned Assets Supervision and Administration Commission urged relevant national enterprises to return to work and quicken the production of prevention and control materials and therapeutic drugs. In Hubei Province, as stated in the news conference on February 22, 2020, the medical protective clothing supply increased from 10 000 up to 170 000 a day, and that of N95 respirator from 36 000 to 300 000 a day. And the public were encouraged to make a donation. The shortage details were released promptly and continually by the local leading group, and the local Red Cross Society was always ready to receive and distribute donations in an open and transparent way. As of April 1, 2020, the public donations received by the Red Cross Society of China amounted to 2104.63 million yuan in total, which was provided for charity in the light of donors’ wishes and supervised by National Audit Office. As of March 4, 2020, the financial appropriation from the governments at all levels reached up to 1 104 800 million yuan, according to the National Ministry of Finance.

All the expenses for COVID-19 treatment of diagnosed and suspected cases were covered by the national medical insurance, and the designated hospitals were prepaid by national medical insurance to dispel patients’ and hospitals’ financial concerns and guarantee their diagnosis and treatment. If a person was put in quarantine for treatment or medical observation because of COVID-19, he/she would not suffer from salary cut or dismissal. Almost all official ministries have done their best to guarantee manpower and material resources.

#### Mobilization of medical workers

Besides local medical personnel, thousands of health workers in other places have been organized or mobilized to go to Hubei Province. As of March 5, 2020, 330 medical teams, consisting of over 40 000 persons from other provinces had been sent to support Hubei for COVID-19 epidemic prevention and control in succession.

Medical workers’ infection with or death of SARS-COV-2 during work must be identified as industrial injury and they would obtain corresponding compensation. They would be additionally paid extra for fighting against COVID-19, free of personal income tax. Their inspiring deeds were recorded and propagated. At the same time, the government put an eye on public functionary. Anyone who neglects his/her own duty or whoops up the price of prevention and control material would get punished.

### Complete activation of national institutions, especially the public health system

As of January 25, a total of 30 provinces activated to level 1 public health emergency response, except Hong Kong, Macao and Taiwan (Table 2). Tibet did not launch level 1 public health emergency response until January 29, right after the diagnosis of the first imported patient with SARS-CoV-2 on January 28. Three provinces, including Hubei, Tibet and Hainan activated level 2 public health emergency response initially, which was then elevated to level 1. Almost all the level 1 public health emergency responses were started up in 0–4 days after the first case diagnosed, or in 0–5 days after the official claim of human-to-human transmission. Guangdong and Zhejiang were the first two provinces coming into level 1 public health emergency response (Table 2). Each province promptly lowered the response level based on the local epidemic. As of June 6, none province was at level 1 public health emergency response, two provinces were at level 2, twenty-six at level 3, and three at level 4. From level 1 to 4, the epidemic situation was becoming increasingly mild.

The government has taken regulations and schemes to guide, standardize or legalize the COVID-19 prevention and control activities, covering all aspects listed in Table 3. Although COVID-19 was classified as category B infectious diseases, management measures were taken according to the criteria for category A infectious diseases.

### Guiding the public to understand and adhere to the practices

Regular press conferences were held. Guiding principles of emergency psychological crisis intervention in the COVID-19 epidemic were issued by the government on January 26, 2020, and 24 h psychological assistance hotline was set up in each province during the COVID-19 epidemic. The public were always taught about some major topics online to offline. Many books about COVID-19 prevention and control were urgently published. And the information was reported to the public at official websites of WHO or local authorities on a daily basis in several languages.

### Others

Ten key field research programs planned to be carried out, so as to provide technical support for the COVID-19 prevention and control. Radiation safety supervision was taken into consideration. The effect of the epidemic on medium-sized and small enterprises was investigated on-line from February 3 until 25, 2020, and they obtained compensation to some extent from the government in the form of rent concession, reduction of electricity and gas fees, tax abatement, deferred payment of medical insurance, social security. Besides, they were encouraged to resume work as long as they met the prevention and control requirements.

### Whole effect of practices on population shift

**Fig. 1:**
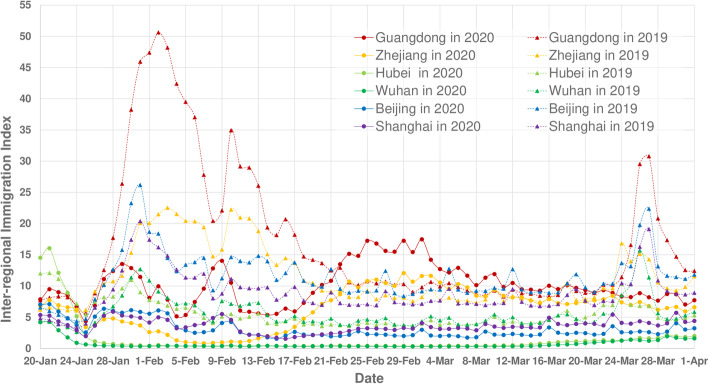
Comparison of inter-regional immigration index in Guangdong, Zhejiang, Hubei, Wuhan, Beijing, and Shanghai between 2019 and 2020. The bigger the immigration index is, the more the population size of immigrating to the areas is. The dates have been adjusted according to Chinese Calendar, so as to make comparisons comparability between 2019 and 2020

**Fig. 2:**
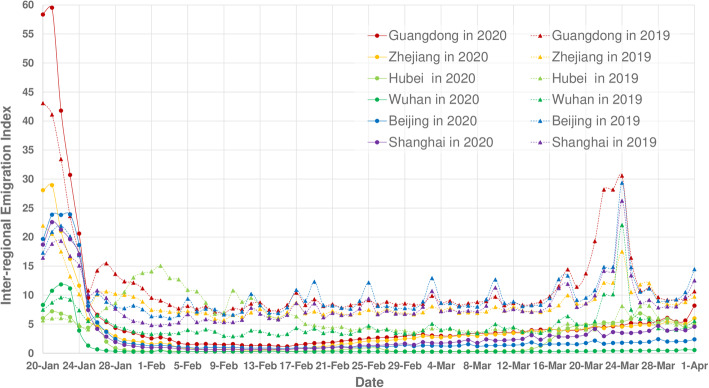
Comparison of inter-regional emigration indexes in Guangdong, Zhejiang, Hubei, Wuhan, Beijing, and Shanghai between 2019 and 2020. The bigger the emigration index is, the more the population size of emigrating from the areas is. The dates have been adjusted according to Chinese Calendar, so as to make comparisons comparability between 2019 and 2020

**Fig. 3:**
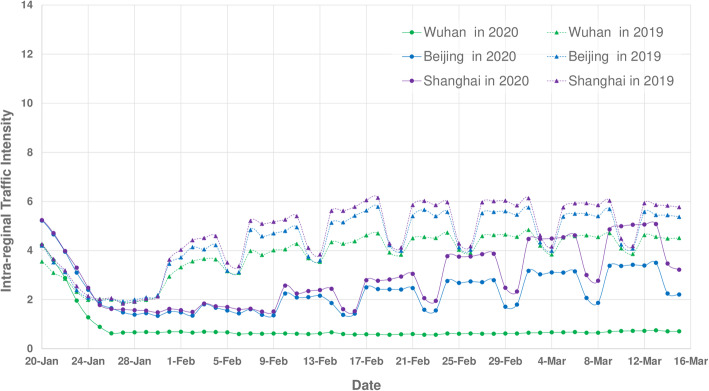
Comparison of intra-regional traffic intensity in Wuhan, Beijing, Shanghai cities between 2019 and 2020. The urban traffic intensity is equal to the indexation result from the ratio of population traveling outside the home to the total population in the city. The dates have been adjusted according to Chinese Calendar, so as to make comparisons comparability between 2019 and 2020

In 2020, the measures to cut off the transmission route exerted a tremendous effect on the population shift level in China, resulting in a continual decline to a low ebb during the Spring Festival. Conversely, the population shift level came into a peak in the same period of 2019 (Additional file 3: Figure S1). The effects were further illuminated by the change trends of inter-regional immigration index, inter-regional emigration index and intra-regional urban traffic intensity in cities of Beijing, Shanghai, Wuhan, and provinces of Hubei and Zhejiang, Guangdong (Figs. 1, 2, 3). The inter-regional immigration index, inter-regional emigration index and intra-regional urban traffic intensities in 2020 were always obviously lower than that in 2019. In Zhejiang or Guangdong, however, there was a period that the inter-regional immigration indexes in 2020 were significantly larger than that in 2019 since February 22 (Fig. 1). Moreover, the population shift level was slowly climbing up since February 10, 2020 nationwide, which may be a consequence of the release of the policies for work and production resumption on February 8, 2020 by the State Council (Table 3, Additional file 3: Figure S1).

### Whole effect of practices on virus transmission

**Fig. 4:**
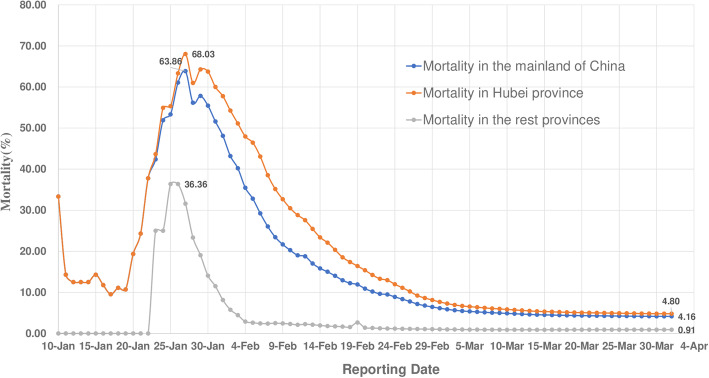
The changing trend of COVID-19 mortalities in the mainland of China, Hubei Province and the rest provinces. The mortality was calculated as follows: [the number of deaths / (the number of deaths + the number of recovered) × 100%]

As of April 1, 2020, a total of 81 589 diagnosed cases (67 802 in Hubei, 13 787 in other areas), 97 895 suspected cases, and 709 570 close contacts had been reported, found out and medically observed in the mainland of China, respectively. China has been doing its best to treat patients. Nevertheless, 3318 patients died at last (3199 in Hubei, 119 in other areas). The overall mortality was around 4.16% in the mainland of China, with 4.80% in Hubei Province and 0.91% in non-Hubei provinces (Fig. 4). At the early period, the mortalities were high up to more than 60% (e.g. 68.03% in Hubei Province), however, with the keeping improvement of measures (e.g. prophylaxis and treatment schemes) based on a clearer understanding about the emerging disease, the overall mortalities stably declined to about 4.16%. The mortalities in the rest provinces (from 36.36 to 0.91%) was much lower than that in Hubei Province (from 68.03 to 4.80%). That’s because the epidemic in Hubei Province was much more serious and medical resources were relatively insufficient compared to that in the rest provinces (Fig. 4). Moreover, the high mortality at the early stage was also due to the small sample of patients which might lead to bias.

**Fig. 5:**
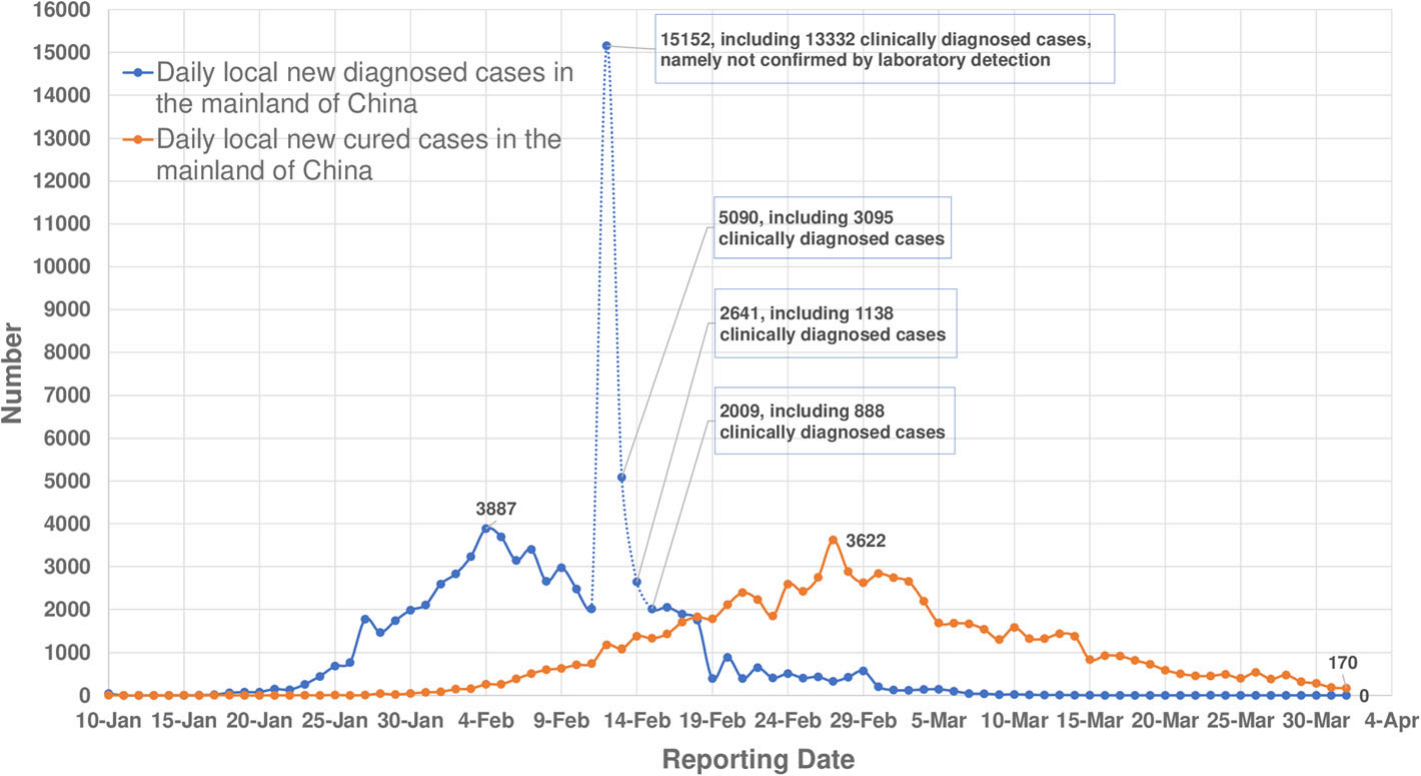
The changing trend of the number of daily local new diagnosed and cured patients in the mainland of China

**Fig. 6:**
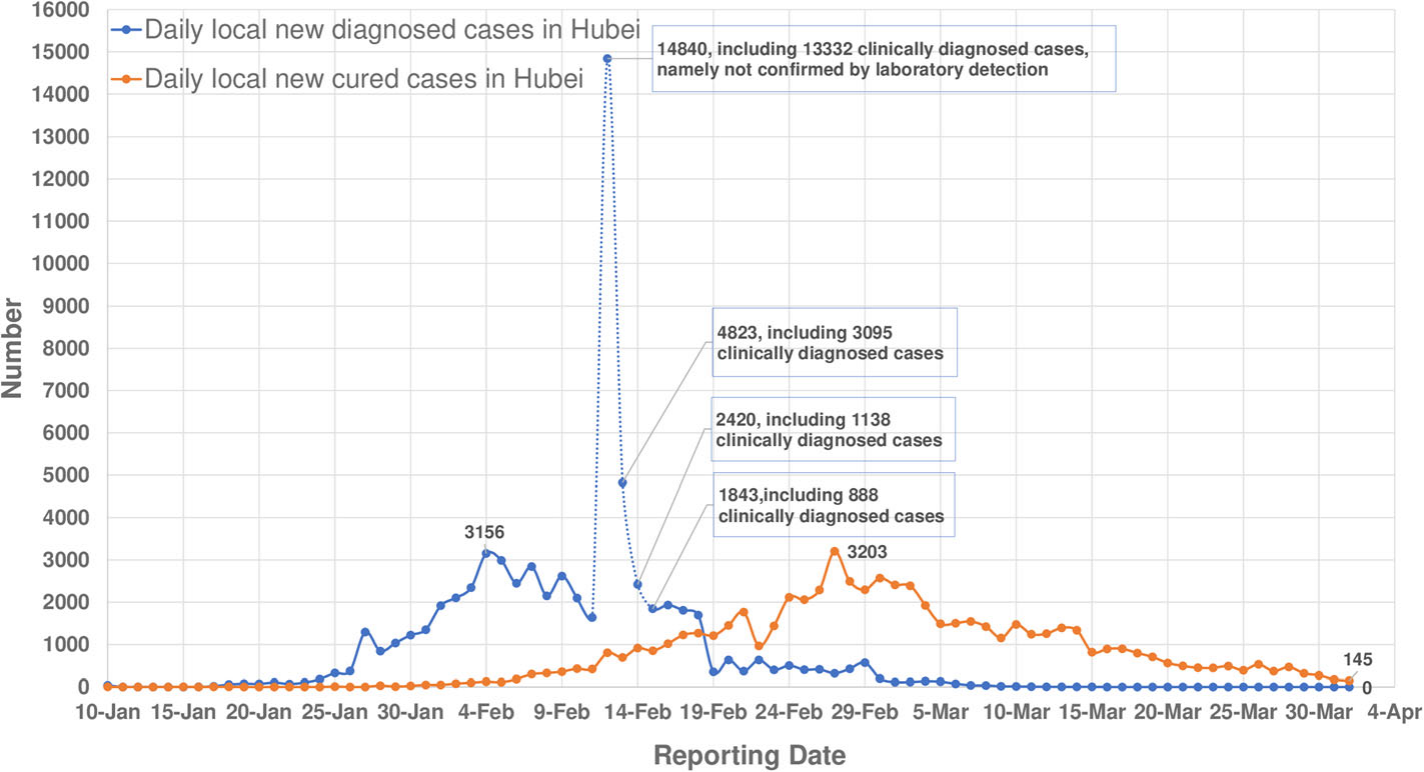
The changing trend of the number of daily local new diagnosed and cured patients in Hubei Province

**Fig. 7:**
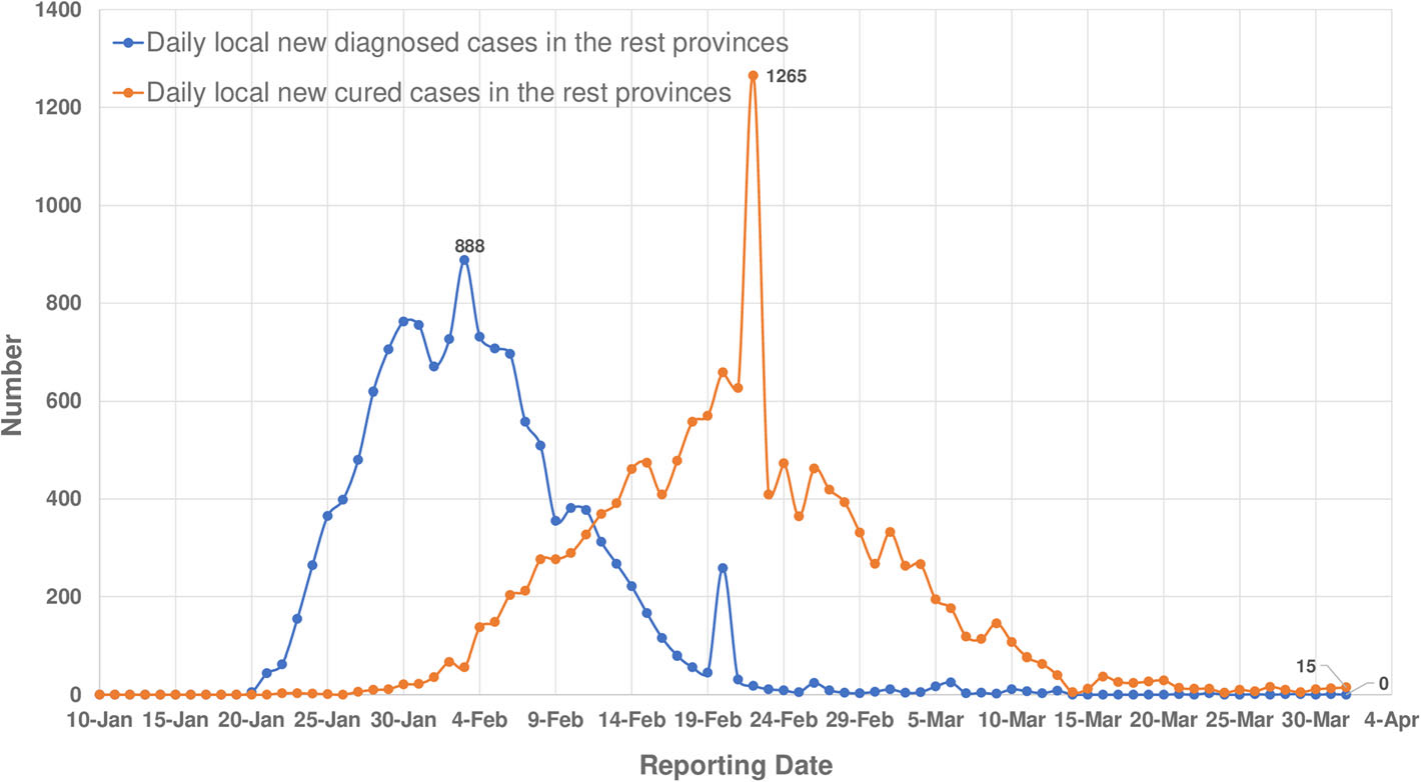
The changing trend of daily local new diagnosed and cured cases in other provinces in China

In China, the overall trend of the number of daily local new diagnosed cases changing with time was kind of irregular, which was resulted from the clinically diagnosed cases. Specifically, it seemed that the daily local new diagnosed cases reached a peak of 3887 on February 4, 2020. Since then, the number of daily local new diagnosed cases started to decrease. However, the clear change trend might be confused due to the report of clinically diagnosed cases from Hubei Province, which were not confirmed by laboratory detection. Since February 19, the number of daily local new diagnosed cases became less than the daily local new cured patients (Fig. 5). In Hubei Province, the trends of the numbers of daily local new diagnosed and cured cases changing with the time were the same with the nationwide figures (Fig. 6). In the rest provinces, the variation trend of the number of daily local new diagnosed cases along with the time was quite regular, which crept up to the apex on February 3, 2020, one day earlier than that in the mainland of China or Hubei Province and then kept declining. And daily local new cured cases outnumbered daily local new diagnosed cases since February 12 (Fig. 7). Moreover, though special attention has been paid to high-risk groups such as nursing homes and prisons, there were still two outbreaks occurred in two prisons, namely “Shilifeng Prison in Zhejiang Province” and “Rencheng Prison in Shandong Province”.

Evidence showed that China had overcome the hardest time of the epidemic. The practices taken to prevent and control COVID-19 were fruitful.

## Discussion

The Chinese government has been doing its best to prevent and control COVID-19 with a high degree of transparency [23]. Since the outbreak of COVID-19, supreme leaders at all levels have laid special stress on the prevention and control, starting up emergency response, setting up work leader groups and panels of consultation and scientific research, so as to ensure the smooth and scientific implementation.

Being faced with a completely new infectious disease, China had taken ever-changing measures to prevent and control the disease, based on the updating understanding of COVID-19, the epidemic progress, related technologies, and national economic strategy (Tables 2 and 3, Additional file 1: Table S1, Additional file 2). We would like to explain the prevention and control process by three stages, as follows: the stage before January 20, 2020 (stage 1), the stage from January 20, 2020 to February 8, 2020 (stage 2), and the stage after February 8, 2020 (stage 3). January 20, 2020 was the first landmark day. Lots of important responses were activated on the day, as below. 1) Human-to-human transmission was confirmed and claimed to the public; 2) the work leader group at the national level was set up, making an overall arrangement for the combat; 3) COVID-19 control was brought into the management of legal infectious diseases, and also included as one of the quarantinable infectious diseases, so that the control activities were guaranteed legally. In our opinion, the combat against SARS-CoV-2 was more forcefully initiated since then. Another milestone event happened on February 8, 2020, with a release by NHC about the resumption of enterprise work and manufacturing during the epidemic.

Some basic prevention and control activities were implemented throughout the period, such as surveillance, epidemiological investigation, quarantine, treatment, and measures for cutting down the transmission route. Different stages varied from each other with the objectives. At the 1st stage when a kind of unexplained pneumonia broke out, the primary task was to make certain the possibility of human-to-human transmission, pathogenic agent, source of infection, transmission route and susceptible population, and clinical and epidemiological features. All of the basic technologies have been accomplished, including pathogen sampling, isolation and RNA sequencing [24], formulating schemes for diagnosis and treatment, prevention and control, laboratory detection, developing detection kit [17, 25–28], and shutting down local wild animal and live poultry markets [17]. Another two key actions, namely reporting to WHO and completely sharing genome sequence, were also promptly taken, which would benefit other countries fighting against the virus [17].

Since January 20, 2020, the combat with SARS-CoV-2 entered stage 2, which was the most critical, remarkable, and difficult period. The whole country plunged into the fighting. One of the strategies in this stage was to prevent cases from spreading between areas. As more and more cases from Wuhan entered other provinces, the city was closed since January 23, 2020, which never happened in the Chinese history. Right after that, up to another 17 cities in Hubei Province were closed too, with one city (Shennongjia) left unsealed. Unfortunately, the epidemic occurred just before the Spring Festival of China, an event that would have the world largest population movements. Thousands of Chinese people go home and soon go back to work or school around the Spring Festival, which extremely makes for inter-or intra-regional transmission of viruses. Therefore, the Chinese government has taken several measures to reduce the population shift. The ending date of the Spring Festival holiday was postponed from January 30 to February 2 and the starting date of schools was put off and undetermined, so as to avoid the travel rush for purpose of resumption of school or work (Figs. 1, 2, 3, Additional file 3: Figure S1).

Moreover, there was no curative drug or vaccine for the disease. The prevention and control largely relied on the measures for cutting down transmission, so as to reduce prevalence strength. The guidance for COVID-19 diagnosis and management, or, prevention and control had been revised and perfected from time to time according to the experience and new knowledge about the virus (Table 3, Additional file 2, Additional file 1: Table S1). Since January 20, 2020, people were suggested to stay at home as far as possible. The entry and exit administration were carried out at ports and in communities, and gathering and visiting during the Spring Festival was forbidden by the government. There were also some people going out for the gathering, and getting infected with SARS-CoV-2 [4, 29–31] finally. Another objective of stage 2 was to improve the cure rate, reducing mortality. During the epidemic, medical resources were continually replenished and enhanced. Specifically, hospital beds were in short supply in Hubei Province initially, and the shortage was completely solved rapid construction of special hospitals, “Leishenshan Hospital”, “Huoshenshan Hospital” and “Fangcang Hospital”.

The economy is always the priority of China under normal circumstances. Besides, the Chinese government has set a goal to realize a well-off society for every citizen by 2020 [32, 33]. Soon after the epidemic is suppressed, it is bound to consider promoting economic development and disease prevention and control simultaneously. COVID-19 prevention and control in stage 3 is more demanding. Several new schemes for COVID-19 prevention and control in enterprises, shopping malls and supermarkets were released, and lots of policies have been taken to encourage resumption of work or production (Table 3). We are convinced that the implementation of new measures on top of those already adopted will lead to good results of prevention and control and allow the return of economic stability and growth.

Up to now, China has gone through the hardest time of combating with the epidemic. With less and less daily local new diagnosed cases in Hubei Province, and almost zero growth in other provinces, it is obvious that the increasing trend of COVID-19, an extremely communicable respiratory disease, has been reversed. The *R*_*0*_ was estimated at about 1.0–5.7 [8, 34–41]. Until April 1, 2020, the total number of patients with COVID-19 was 81 589, which was hundreds of thousands less than the estimated [36, 37], proving the effectiveness of China’s practices [42].

However, it is a pity that the current study cannot make too much analysis from the point of view of epidemiology, public health or epidemic system. Measures recorded in this paper would go a long way toward understanding the broad application of these public health interventions. Further studies are waiting to carry out, on how a control measure (e.g. announcement of travel routes of confirmed cases) used in China play an important role in combating against the COVID-19 epidemic and on if the control measure would apply to the other countries.

## Conclusions

China’s practices (involving active case surveillance, rapid case diagnosis and quarantine, strict follow-up and quarantine of close contacts and issuance of guidance to help the public to understand and adhere to control measures), plus prompt and effective high-level policy decision, complete activation of the public health system, and full involvement of the society, are effective to prevent and control COVID-19. Considering the new current situation (e.g. imported cases from abroad, work resumption), China needs to promptly strengthen and adjust the measures.

## Supplementary information

**Additional file 1: Table S1.** The management measures differences amongst the versions of guidance for COVID-19 prevention and control.

**Additional file 2.** Text. The meaning of each symbol in Table 1 and the diagnostic criteria differences amongst the versions of guidance for COVID-19 diagnosis and management.

**Additional file 3: Figure S1.** Comparison of national migration index in the mainland of China between 2019 and 2020. The bigger the migration index is, the more the population flow of migration among cities is. The dates have been adjusted according to Chinese Calendar, so as to make comparisons comparability between 2019 and 2020.

**Additional file 4.** The original data of daily mortality.

**Additional file 5.** The original data of population shift levels.

**Additional file 6.** The original data of daily local new diagnosed and cured cases.

## Data Availability

All data supporting the findings of this study are included in the article.

## Abbreviations

COVID-19: Coronavirus disease 2019
SARS-CoV-2: Severe acute respiratory syndrome coronavirus 2
NHC: National Health Commission of the People’s Republic of China
