# Supplementary material for: China’s practice to prevent and control COVID-19 in the context of large population movement
Source: Infect Dis Poverty. 2020 Aug 19;9:115. doi: 10.1186/s40249-020-00716-0 (PMC7435224; doi:10.1186/s40249-020-00716-0)
Supplement: Supplementary file 1 — Additional file 1: Table S1. The management measures differences amongst the versions of guidance for COVID-19 prevention and control. [file 40249_2020_716_MOESM1_ESM.doc]

**Appendix Table 1. The management differences amongst** **the versions of guidance for COVID-19 prevention and control.**

| **Version** | **Inspected case** | **Laboratory or Clinically* diagnosed case** | **Asymptomatic infection** | **Close contact** |
| --- | --- | --- | --- | --- |
| 1 | reporting in 2h,  epidemiological survey in 24h,  sampling and detection,  quarantine and treatment. | reporting in 2h,  quarantine and treatment. | - | home or centered, isolation, medical observation for 14d. |
| 2 | reporting in 2h,  epidemiological survey in 24h,  sampling and detection,  quarantine alone and treatment. | reporting in 2h,  epidemiological survey in 24h,  **quarantine and treatment, as soon as possible for critical type**. | - | home or centered isolation, medical observation for 14d. |
| 3 | reporting in 2h,  epidemiological survey in 24h,  sampling and detection,  quarantine alone and treatment. | reporting in 2h,  epidemiological survey in 24h,  quarantine and treatment, as soon as possible for critical type, or **home isolation, treating for mild type during resource shortage stage.** | **reporting in 2h,**  **epidemiological survey in 24h, home isolation, treating, observing.** | home or centered isolation medical observation for 14d. |
| 4 | reporting in 2h,  epidemiological survey in 24h,  sampling and detection,  quarantine alone and treatment. | reporting in 2h,  epidemiological survey in 24h,  quarantine and treatment, as soon as possible for critical type, **quarantine alone for clinical diagnosis**. | reporting in 2h,  epidemiological survey in 24h, **centered isolation for 14d or 7d once detection negative**. | home or centered isolation medical observation for 14d. |
| 5 | reporting in 2h,  epidemiological survey in 24h,  sampling and detection,  quarantine alone and treatment. | reporting in 2h,  epidemiological survey in 24h  quarantine and treatment, as soon as possible for critical type. | reporting in 2h,  epidemiological survey in 24h, **centered isolation for 14d with two times negative detection**. | home or centered isolation medical observation for 14d. |
| 6 | reporting in 2h,  epidemiological survey in 24h,  sampling and detection,  quarantine alone and treatment. | reporting in 2h,  epidemiological survey in 24h,  quarantine alone and treatment. | reporting in 2h,  epidemiological survey in 24h, centered isolation for 14d with two times negative detection. | home or centered isolation medical observation for 14d. |

Note: the data updated to Apr. 1, 2020; “-”: means not getting involved; “*” means the clinical diagnosis only used in Hubei province during the period from Feb. 5 to Feb. 18, 2020, and the clinically diagnosed cases also need separate quarantine and treatment, just as the inspected cases do. The difference between the revised and previous versions is remarked by bold font.
