# Supplementary material for: China’s practice to prevent and control COVID-19 in the context of large population movement
Source: Infect Dis Poverty. 2020 Aug 19;9:115. doi: 10.1186/s40249-020-00716-0 (PMC7435224; doi:10.1186/s40249-020-00716-0)
Supplement: Supplementary file 2 — Additional file 2. Text. The meaning of each symbol in Table 1 and the diagnostic criteria differences amongst the versions of guidance for COVID-19 diagnosis and management. [file 40249_2020_716_MOESM2_ESM.doc]

**Appendix Text**

**Appendix Table 2. Diagnostic criteria differences amongst the versions of guidance for COVID-19 diagnosis and management.**

| **Version** | **Inspected case** | **Laboratory diagnosed case** | **Clinically* diagnosed case** | **Critical**  **type** | **Severe**  **type** | **General**  **type** | **Mild**  **type** | **Recovery**  **type** |
| --- | --- | --- | --- | --- | --- | --- | --- | --- |
| 1 | Ⅰ+(a+b+c+d) | Ⅰ+(a+b+c+d)+(1) | - | f/g/h | - | - | - | - |
| 2 | Ⅱ+(a+b+c) | Ⅱ+(a+b+c)+(2) | - | f/g/h | k/l/m/n/o/p/q/r | - | - | ① |
| 3 | Ⅱ+(a+b+c) | Ⅱ+(a+b+c)+(2) | - | h/i/j | k/m/n/r/s | - | - | ① |
| 4 | Ⅲ+(a+b)/Ⅲ+(a+c)/ Ⅲ+(b+c) | Ⅲ+(a+b)+(3)/Ⅲ+(a+c) +(3)/Ⅲ+(b+c)+(3) | - | h/i/j | k/s/m | b+e | - | ① |
| 5 | For Hubei province:  Ⅳ/(c+e)  For the rest:  Ⅳ+(b+e)/ Ⅳ+(c+e)/ Ⅳ+(b+c)/(b+c+e) | For Hubei province:  Ⅳ+(3)/(c+d)+(3)/ Ⅳ+b+(3)/(c+d)+Ⅱ+(3)  For the rest:  Ⅳ+(b+e)+(3)/ Ⅳ+(c+e)+(3)/ Ⅳ+(b+c)+(3)/(b+c+e)+(3) | Ⅳ+b/(b+c+e) | h/i/j | k/s/m | b+e | e | ①+② |
| 6 | Ⅳ+(b+e)/ Ⅳ+(c+e)/ Ⅳ+(b+c)/(b+c+e) | Ⅳ+(b+e)+(4)/ Ⅳ+(c+e)+(4)/ Ⅳ+(b+c)+(4)/(b+c+e)+(4) | - | h/i/j | k/s/t/u | b+e | e | ①+② |
| 7 | Ⅳ+(b+e)/ Ⅳ+(c+e)/ Ⅳ+(b+c)/(b+c+e) | Ⅳ+(b+e)+(5)/ Ⅳ+(c+e)+(5)/ Ⅳ+(b+c)+(5)/(b+c+e)+(5) | - | h/i/j | For adults:  k/s/t/u  For children:  v/w/x/y/z | b+e | e | ①+② |
| # | Ⅴ+(b+e)/ Ⅴ+(c+e)/ Ⅴ+(b+c)/(b+c+e) | Ⅴ+(b+e)+(5)/Ⅴ+(c+e)+(5)/ Ⅴ+(b+c)+(5)/(b+c+e)+(5) | - | - | - | - | - | ①+② |

Note: #: it refers to guidance for COVID-19 prevention and control (Version 6), because the guidance for COVID-19 diagnosis and management has not been revised. The meaning of each symbol is as shown as follows; “+” means “and”; “/” means “or”; “-” means not being involved; “*” means the diagnosis type only used in Hubei province during the implementation period of version 5, which was canceled upon the next version was issued.

The difference between the revised and previous versions is remarked by bold font. As of April 1, 2020, the guidance for COVID-19 diagnosis and management had been revised for 7 times. Based on all the versions, the descriptions of contact history, clinical manifestations, laboratory diagnostic techniques, release of isolation are included as follows.

Contact histories

Ⅰ. Have the history of stay in Wuhan city or direct / indirect contact with markets in Wuhan city, especially agricultural products market within 14 days before the onset of the disease;

Ⅱ. Have the history of stay in Wuhan, or **the history of contact with patients with fever or respiratory symptoms from Wuhan** within 14 days before the onset of the disease or is one of the cluster onsets;

Ⅲ. Have the history of stay in Wuhan or **other places with continual local case**, or the history of contact with patients with fever or respiratory symptoms from Wuhan or **the other places with continual local case** within 14 days before the onset of the disease, or is one of the cluster onsets, or **is epidemiologically associated with SARS-COV -2 infected patients**;

Ⅳ. Within 14 days before the onset of the disease, have the exposure history to Wuhan and its surrounding areas, or **the history of stay in the community with SARS-COV-2 infected patients**; or have the history of contact with SARS-COV-2 positive patients within 14 days before the onset of the disease; or have the history of contact with patients with fever or respiratory symptom from Wuhan and its surrounding areas, or **case-positive communities**; or is one of the cluster onsets.

Ⅴ. Within 14 days before the onset of the disease, have the exposure history to Wuhan and its surrounding areas, or the history of stay in the community with SARS-COV-2 infected patients, or **other countries/areas with severe endemic**; or have the history of contact with SARS-COV-2 positive patients within 14 days before the onset of the disease; or have the history of contact with patients with fever or respiratory symptom from Wuhan and its surrounding areas, or case-positive communities, or **other countries/areas with severe endemic**; or is one of the cluster onsets.

Clinical manifestations

a. Fever

b. Imaging features of SARS-COV-2 pneumonia;

c. White blood cell count is normal or has dropped, and the number of lymphocytes has dropped.

d. No significant improvement or even worsening after 3 days of antibacterial treatment;

e. Fever and/or respiratory symptom.

f. Respiratory failure

g. Septic shock

h. Combined with other organ failures and need ICU monitoring and nursing

i. Respiratory failure and need mechanical ventilation

j. Shock

k. Respiratory rate (RR) ≥30/min, dyspnea, cyanosis of lips or when at rest;

l. Oxygen saturation≤95%;

m. Arterial partial pressure of oxygen (PaO_2_)/ fraction of inspired oxygen (FiO_2_) ≤300mmHg;

n. Pulmonary imaging shows multilobar lesion or lesion progression ≥50% in 48h;

o. Quick Sequential Organ Failure Assessment (qSOFA) ≥2

p. Community-acquired pneumonia score ≥1;

q. Combined with pneumothorax;

r. Any other symptoms that need hospitalization

s. Oxygen saturation≤93%,

t. Arterial partial pressure of oxygen (PaO_2_)/ fraction of inspired oxygen (FiO_2_) ≤300mmHg, adjusted to PaO2/FiO_2_* Atmospheric pressure (mmHg)/760 when the altitude is≥1000 meters.

u. Pulmonary imaging shows lesion progression ≥50% in 24~48h;

v. At the time when not being affected by fever and crying, suffering from shortness of breath (<2 months old, RR ≥60/min; 2~12 months old, ≥50/min; 1~5 years old, ≥40/min; >5 years old, ≥30/min);

w. Oxygen saturation≤92% at rest;

x. Somnolence and faint from fear;

y. Antifeeding or feeding difficulties, and dehydration;

z. Moan, nasal ale flap, three depression signs, cyanosis, intermittent apnea.

Laboratory diagnostic techniques

(1) Virus nucleic acid sequences isolated from the respiratory tract samples (sputum or throat swab) are highly homologous to SARS-COV-2.

(2) Using the samples of sputum, throat swab, or lower respiratory secretion, **SARS-CoV-2 nucleic acid detection by RT-PCR shows positive** or the sequencing results show that the pathogen is highly homologous to SARS-COV-2;

(3) Using the respiratory tract or **blood samples**, SARS-CoV-2 nucleic acid detection by RT-PCR shows positive or the sequencing results show that the pathogen is highly homologous to SARS-COV-2;

(4) Using **the samples** from patients, SARS-CoV-2 nucleic acid detection by RT-PCR shows positive or the sequencing results show that the pathogen is highly homologous to SARS-COV-2, or **results show that SARS-COV-2 IgM and IgG antibody are positive in serum, or it is found that SARS-COV-2 IgG antibody changes from negative to positive or four-fold elevation in acute phase serum over that in convalescent phase serum.**

Release of isolation and discharge criteria

1. The body temperature is normal for more than 3 days, and respiratory symptoms are significantly recovered, with negative results of SARS-COV-2 nucleic acid detection for consecutive two times (sampling interval is at least 1 day);
2. Lung imaging shows obvious absorption and recovery of acute exudative lesions.

As of March 18, 2020, the guidance for COVID-19 prevention and control had been revised for 6 times. Based on all the version 2-5, criteria for close contact are as showed in “ⅱ”, version 6 as in “ⅲ”.

ⅱ. Close contacts refer to people who have contacted with suspected or diagnosed cases after disease onset, or positive asymptomatic carriers, and satisfy one of the following situations, but have not taken effective protection:

Living, studying or working together, or having close contacts in other situations, such as working at close range or sharing same classrooms or living in same house;

Medical staff who provide diagnosis and treatment services to patients, or family members who provide care or visit, or anyone who have similar close contact with cases, such as visiting or staying in a confined environment, or other patients and their accompanying staff in the same ward;

People who are in same transportation and have close contact with cases, including cares or accompanying persons (family members, colleagues, friends, etc.) on the same transportation, or other passengers or flight attendants who may have close contact with cases or asymptomatic carriers after investigation.

People who are considered satisfying as close contacts criteria after field investigation and evaluation.

ⅲ. **People have close contact with suspected or diagnosed cases 2-day before disease onset, or with positive asymptomatic carriers 2-day before sampling, but have not taken effective protection.**
