# Supplementary figures and images for: China’s practice to prevent and control COVID-19 in the context of large population movement
Source: Infect Dis Poverty. 2020 Aug 19;9:115. doi: 10.1186/s40249-020-00716-0 (PMC7435224; doi:10.1186/s40249-020-00716-0)

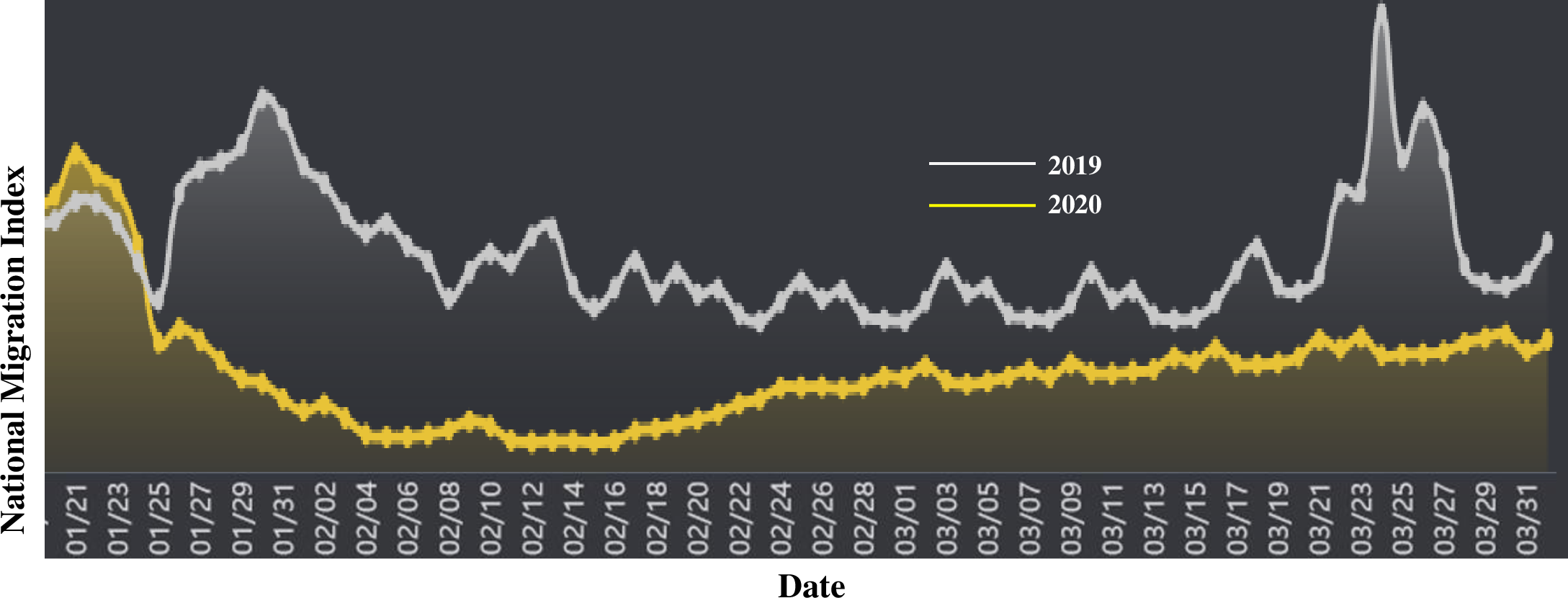

Supplement: Supplementary file 3 — Additional file 3: Figure S1. Comparison of national migration index in the mainland of China between 2019 and 2020. The bigger the migration index is, the more the population flow of migration among cities is. The dates have been adjusted according to Chinese Calendar, so as to make comparisons comparability between 2019 and 2020. [file 40249_2020_716_MOESM3_ESM.tif]
